# Supplementary material for: Robust regulatory interplay of enhancers, facilitators, and promoters in a native chromatin context
Source: bioRxiv. 2025 Jul 9:2025.07.07.663560. Preprint. [Version 1] doi: 10.1101/2025.07.07.663560 (PMC12265581; doi:10.1101/2025.07.07.663560)
Supplement: Supplement 5 [file NIHPP2025.07.07.663560v1-supplement-5.pdf]

bioRxiv preprint doi: https://doi.org/10.1101/2025.07.07.663560; this version posted July 9, 2025. The copyright holder for this preprint (which was not certified by peer review) is the author/funder, who has granted bioRxiv a license to display the preprint in perpetuity. It is made available under aCC-BY 4.0 International license.

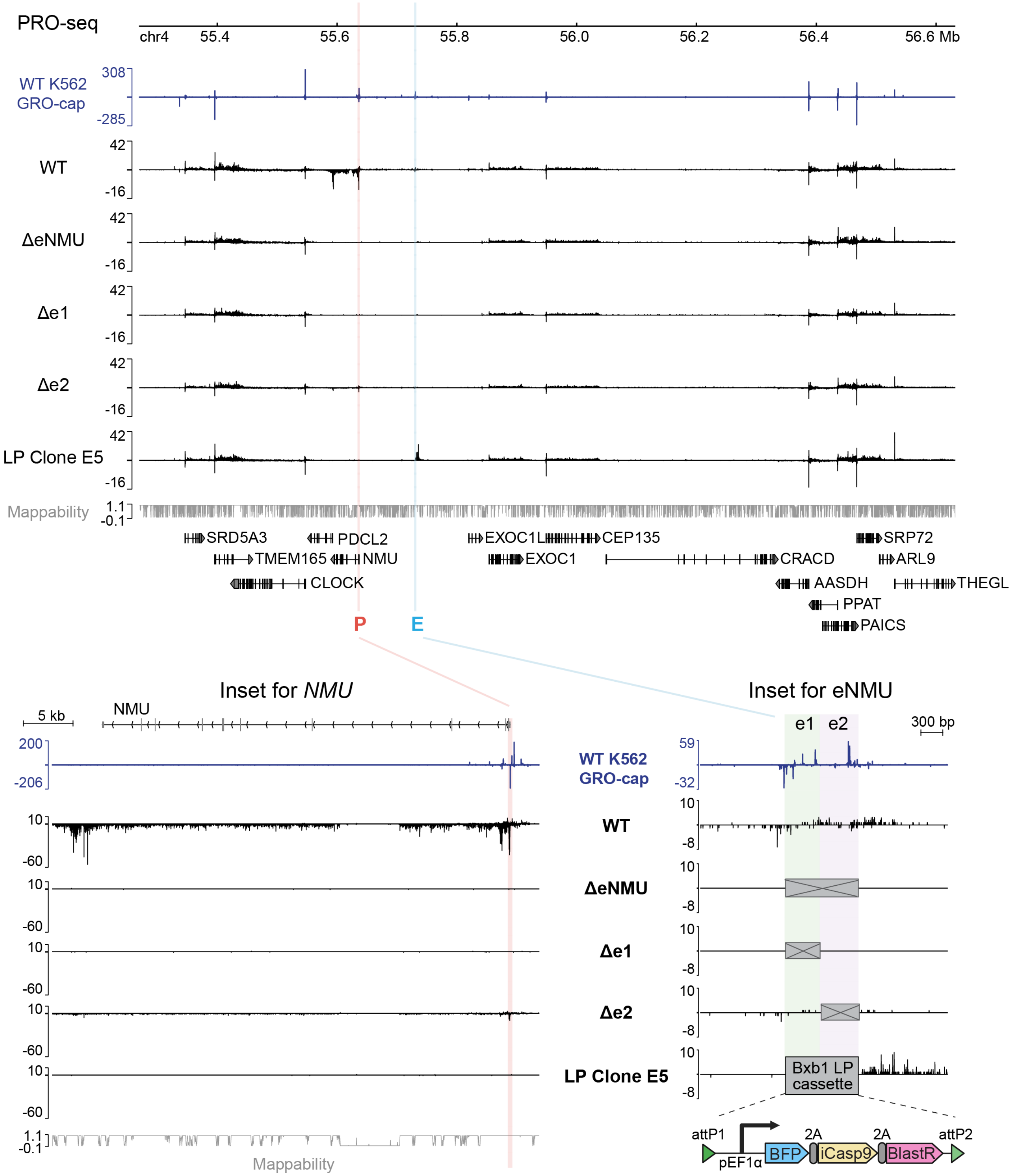

bioRxiv preprint doi: https://doi.org/10.1101/2025.07.07.663560; this version posted July 9, 2025. The copyright holder for this preprint (which was not certified by peer review) is the author/funder, who has granted bioRxiv a license to display the preprint in perpetuity. It is made available under aCC-BY 4.0 International license.

**Figure S1. eNMU specifically regulates *NMU* gene transcription in K562**

PRO-seq (3′-end) tracks of WT, ΔeNMU, Δe1, Δe2 and eNMU LP cell lines across a ~1 Mb region around *NMU*; insets show the full *NMU* gene and eNMU loci. Highlighted regions indicate *NMU* promoter (P) and eNMU (E). Note that at the eNMU locus in LP Clone E5, the prominent PRO-seq signal downstream (to the right) of the Bxb1 LP cassette originates from readthrough transcription driven by the strong EF1α promoter within the selection cassette. However, transcriptional activity of the LP did not exhibit any enhancer function to activate the distal *NMU* gene. Tracks represent merged biological replicates (n = 2 independent cultures). WT K562 GRO-cap data[8] shown as the TSS reference.

Related to Figure 1.

bioRxiv preprint doi: https://doi.org/10.1101/2025.07.07.663560; this version posted July 9, 2025. The copyright holder for this preprint (which was not certified by peer review) is the author/funder, who has granted bioRxiv a license to display the preprint in perpetuity. It is made available under aCC-BY 4.0 International license.

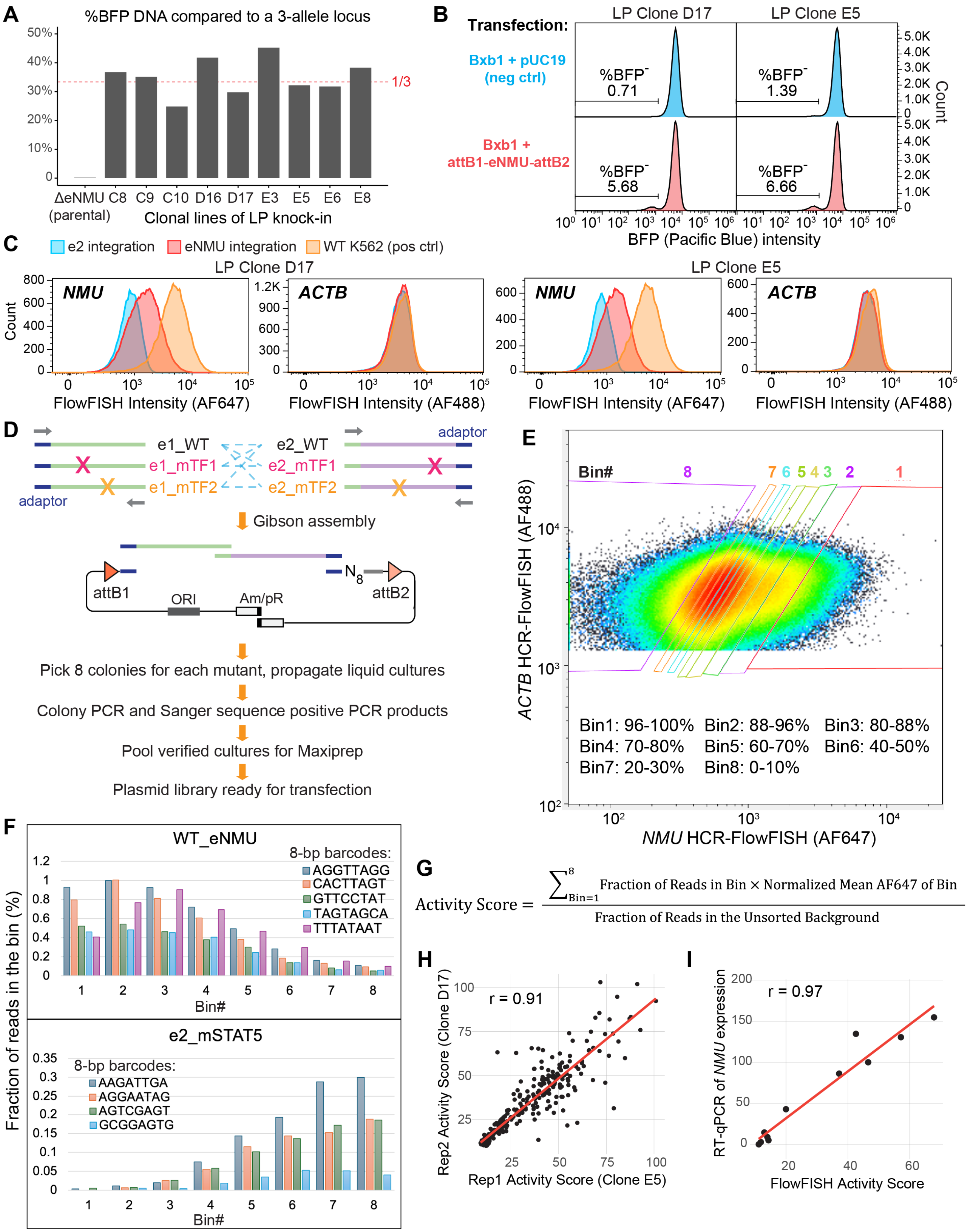

bioRxiv preprint doi: https://doi.org/10.1101/2025.07.07.663560; this version posted July 9, 2025. The copyright holder for this preprint (which was not certified by peer review) is the author/funder, who has granted bioRxiv a license to display the preprint in perpetuity. It is made available under aCC-BY 4.0 International license.

## Figure S2. eNMU landing pad construction and mutant screen

(A) LP copy number analysis by quantitative PCR on genomic DNA (BFP DNA vs. 3-allele control locus).
(B) Bxb1 recombination efficiency measured by BFP loss in two independent LP clones using flow cytometry.
(C) Validation of HCR-FISH in the same LP clones as in (B); *ACTB* served as the housekeeping gene control.
(D) Gibson assembly workflow for constructing the barcoded eNMU mutant library.
(E) Flow cytometry binning strategy using *ACTB* as an internal control for cell size, transcription level, and staining efficiency.
(F) Barcode distribution across 8 sorting bins for two example elements in the mutant library: WT_eNMU and e2_mSTAT5.
(G) Calculation of activity scores using a weighted average of barcode distributions.
(H) Correlation of median barcode activity scores between biological replicates (n = 2 independent LP clones subjected to recombination and FlowFISH). Pearson's correlation coefficient (r) is shown.
(I) Correlation between FlowFISH-measured activity scores and RT-qPCR quantifications of select mutants, based on median activity values from each assay. Pearson's correlation coefficient (r) is shown.

Related to Figures 1 and 2. See also Methods.

bioRxiv preprint doi: https://doi.org/10.1101/2025.07.07.663560; this version posted July 9, 2025. The copyright holder for this preprint (which was not certified by peer review) is the author/funder, who has granted bioRxiv a license to display the preprint in perpetuity. It is made available under aCC-BY 4.0 International license.

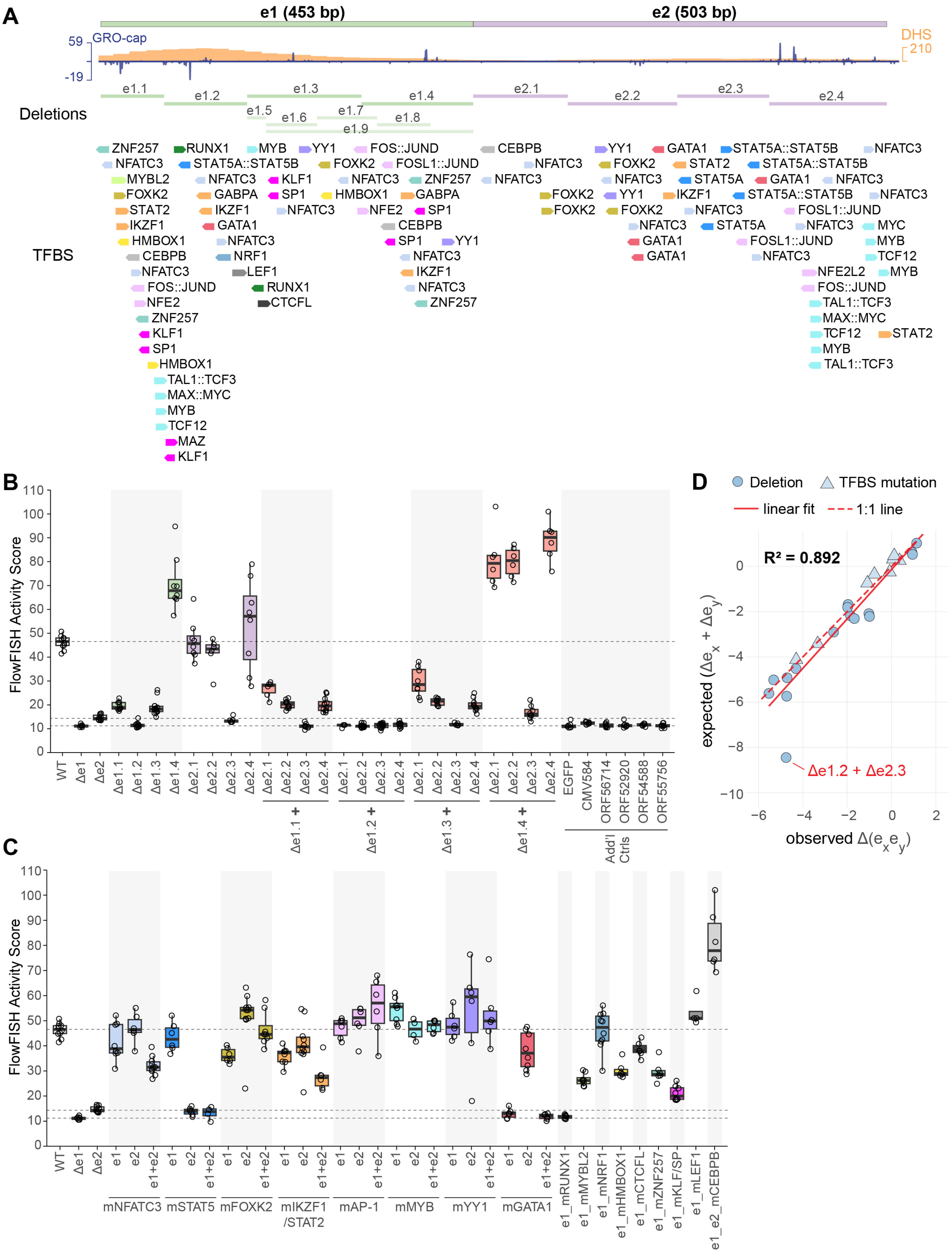

bioRxiv preprint doi: https://doi.org/10.1101/2025.07.07.663560; this version posted July 9, 2025. The copyright holder for this preprint (which was not certified by peer review) is the author/funder, who has granted bioRxiv a license to display the preprint in perpetuity. It is made available under aCC-BY 4.0 International license.

**Figure S3. Complete eNMU mutant screen results reveal a multiplicative model of double mutants' effects**

(A) Full eNMU mutant design separating overlapping motifs of different TFs.

(B) FlowFISH-measured activity scores of single and double deletions, together with additional exogenous control elements.

(C) FlowFISH-measured activity scores of all TF motif mutations in e1, e2, or both. Note that the e1_e2_mCEBPB mutant exhibited higher enhancer activity than WT_eNMU, which may be attributed to an LTR promoter-mediated mechanism as illustrated in Figure 7.

(D) Linear regression of observed $\log_2$ fold changes in double mutants vs. expected additive effects (sum of $\log_2$ fold changes from corresponding single mutants). $R^2$ from linear regression is shown. Dashed 1:1 line indicates perfect additivity. Highlighted outlier: expected effect of the Δe1.2+Δe2.3 mutant fell below FlowFISH's detection range, preventing assessment of additivity.

Related to Figure 2. See also Methods, Tables S1 and S3.

bioRxiv preprint doi: https://doi.org/10.1101/2025.07.07.663560; this version posted July 9, 2025. The copyright holder for this preprint (which was not certified by peer review) is the author/funder, who has granted bioRxiv a license to display the preprint in perpetuity. It is made available under aCC-BY 4.0 International license.

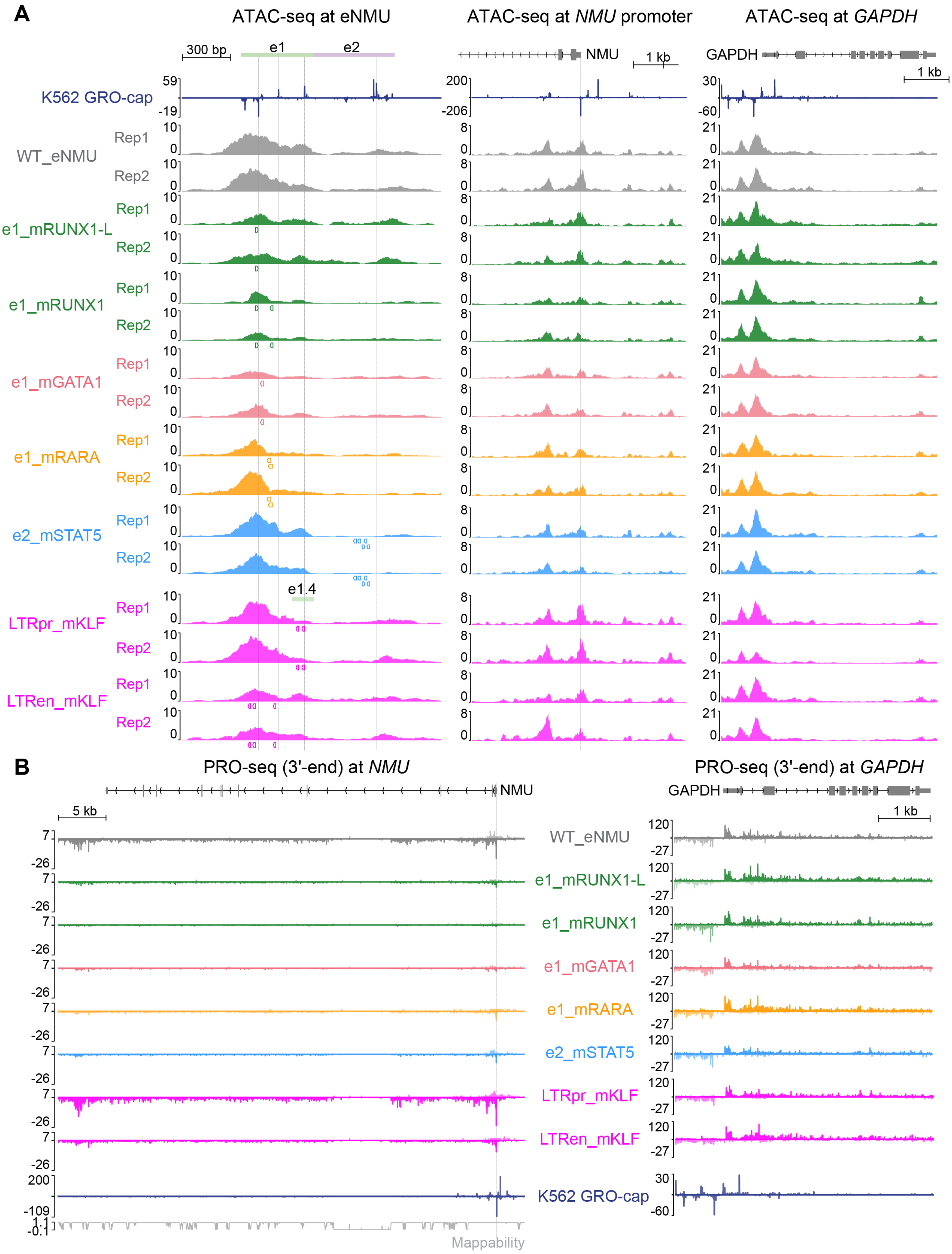

bioRxiv preprint doi: https://doi.org/10.1101/2025.07.07.663560; this version posted July 9, 2025. The copyright holder for this preprint (which was not certified by peer review) is the author/funder, who has granted bioRxiv a license to display the preprint in perpetuity. It is made available under aCC-BY 4.0 International license.

**Figure S4. Reproducibility of ATAC-seq and PRO-seq data across clones and mutants**
(A) ATAC-seq signal at eNMU, *NMU* promoter and *GAPDH* control locus for two independent single cell-derived clones of all analyzed eNMU mutants in this study. Colored boxes below tracks indicate locations of disrupted TF motifs.
(B) PRO-seq tracks at the full *NMU* and *GAPDH* (control) genes in the same mutants as in (A). Tracks represent merged biological replicates (n = 2 independent single cell clones).
Fine vertical lines indicate positions of GRO-cap–defined TSSs (WT K562).[8]

Related to Figures 4 and 7.

bioRxiv preprint doi: https://doi.org/10.1101/2025.07.07.663560; this version posted July 9, 2025. The copyright holder for this preprint (which was not certified by peer review) is the author/funder, who has granted bioRxiv a license to display the preprint in perpetuity. It is made available under aCC-BY 4.0 International license.

**A**

| dTRE | chr | start | end | Target Gene | Dist (kb) | CRISPR perturbation | Readout | Effect Size | Reference |
|---|---|---|---|---|---|---|---|---|---|
| eALAS2 | chrX | 55027939 | 55028537 | ALAS2 | 2.8 | Cas9 deletion of GATA1 motif | RT-qPCR | -93% | Wakabayashi & Ulirsch et al., 2016 |
| HS2 | chr11 | 5280458 | 5281107 | HBE1 | 10.6 | dCas9-KRAB | RNA-seq | -86.48% | Thakore et al., 2015 |
| eCCDC26 | chr8 | 129581969 | 129582705 | CCDC26 | 7.1 | KRAB-dCas9 screen | FlowFISH | -91.86% | Fulco & Nasser et al., 2019 |
| eMYB | chr6 | 135323107 | 135323599 | MYB | 142.1 | dCas9-KRAB screen | RNA-seq | -89.78% | Liu & Horlbeck et al., 2017 |
| NMU e1 | chr4 | 55729890 | 55730343 | NMU | 93.8 | Cas9 deletion | RT-qPCR | -99.98% | Tippens & Liang et al., 2020 |
| eNFE2 | chr12 | 54304240 | 54304761 | NFE2 | 3.5 | KRAB-dCas9 screen | FlowFISH | -89.26% | Fulco & Nasser et al., 2019 |
| eCD164 | chr6 | 109304178 | 109305119 | CD164 | 78.1 | Cas9 small deletion of SNP | RT-qPCR | -65.75% | Ulirsch & Nandakumar et al., 2016 |
| ePIM1 | chr6 | 37183026 | 37183655 | PIM1 | 13.2 | dCas9-KRAB screen | scRNA-seq | -88.88% | Xie et al., 2017 |
| MYC E2 | chr8 | 127960082 | 127960895 | MYC | 224.4 | KRAB-dCas9 screen | FlowFISH | -63.59% | Fulco & Nasser et al., 2019 |

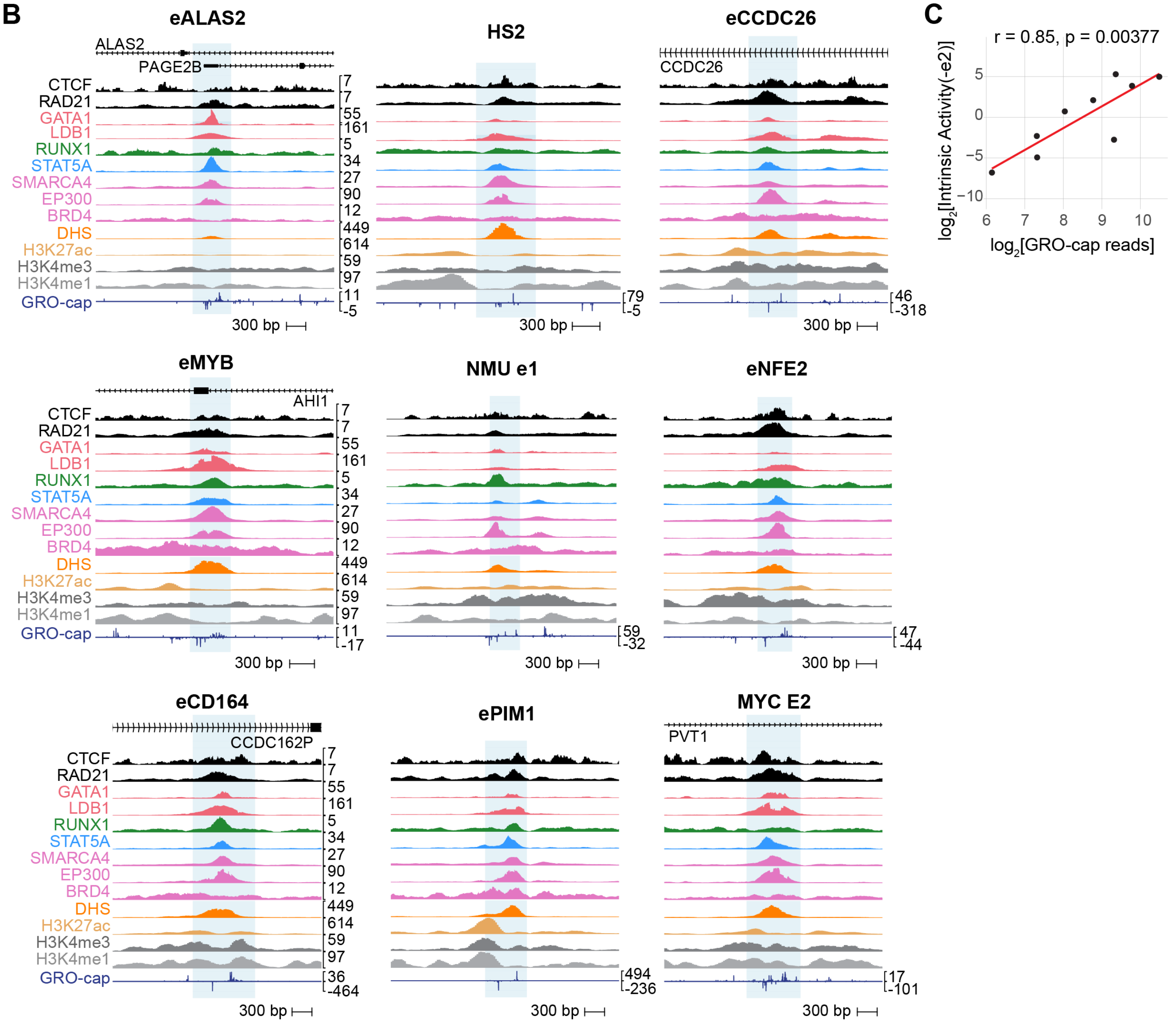

bioRxiv preprint doi: https://doi.org/10.1101/2025.07.07.663560; this version posted July 9, 2025. The copyright holder for this preprint (which was not certified by peer review) is the author/funder, who has granted bioRxiv a license to display the preprint in perpetuity. It is made available under aCC-BY 4.0 International license.

**Figure S5. Testing CRISPR-validated heterologous K562 dTREs at the eNMU locus**

(A) Summarized information of selected K562 dTREs from previous studies. Effect sizes obtained from Fulco et al.[16] (except for NMU e1, which is from Tippens and Liang et al.[9]).

(B) Native genomic contexts of each dTRE; tested regions highlighted in light blue. Track scales are consistent across dTRE regions, except for GRO-cap,[8] which uses an individually indicated scale. Detailed sources and accession information are provided in Table S4.

(C) Correlation between GRO-cap read counts at dTREs and their intrinsic enhancer activity (−e2) at the eNMU locus. Pearson's correlation coefficient (r) and corresponding p-value are shown.

Related to Figure 5.

bioRxiv preprint doi: https://doi.org/10.1101/2025.07.07.663560; this version posted July 9, 2025. The copyright holder for this preprint (which was not certified by peer review) is the author/funder, who has granted bioRxiv a license to display the preprint in perpetuity. It is made available under aCC-BY 4.0 International license.

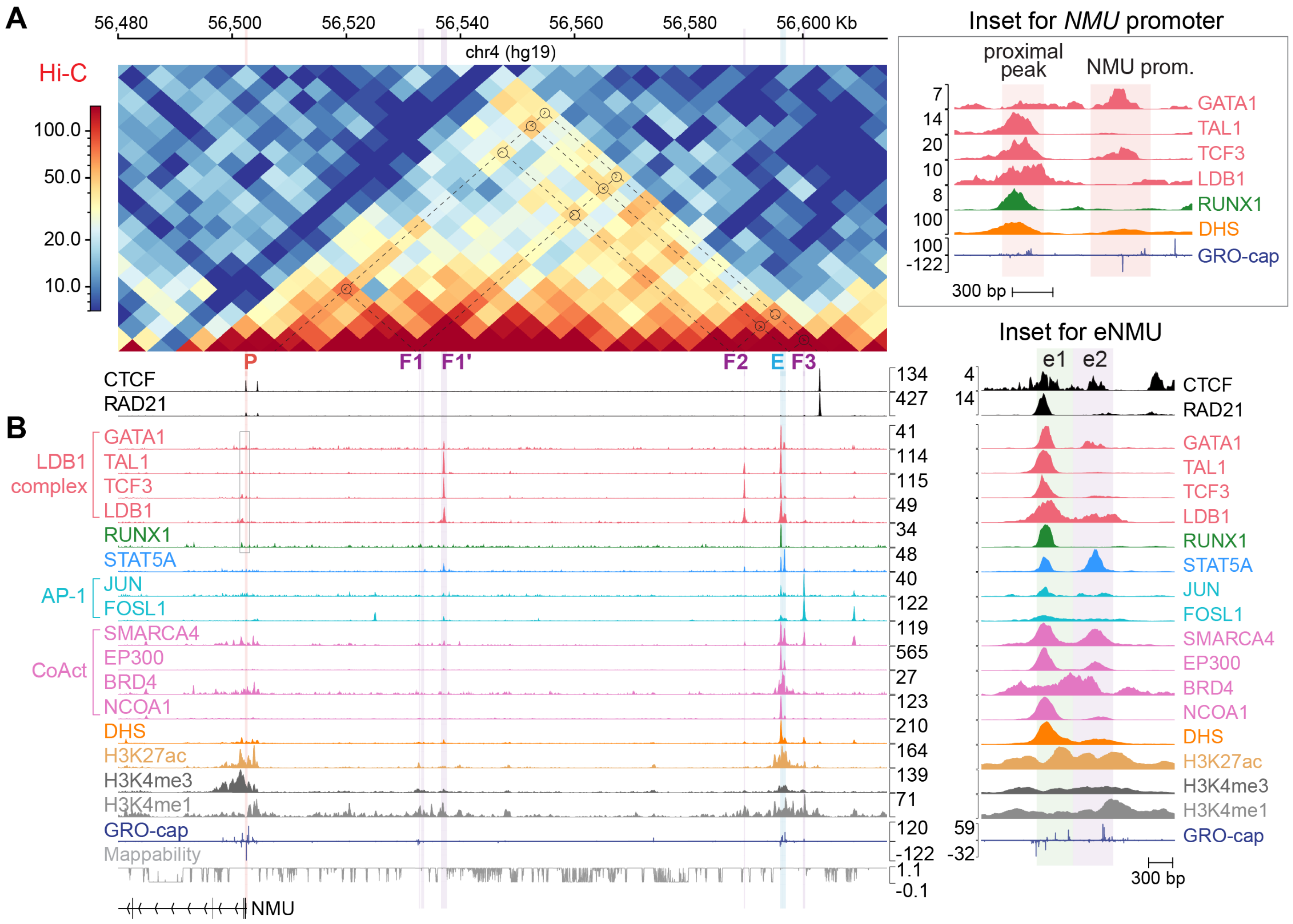


**Figure S6. Additional epigenomic features of enhancer, promoter and facilitators**

(A) Public Hi-C[43] and ChIP-seq tracks[3] of CTCF and RAD21 at the *NMU*–eNMU locus in K562. Hi-C is shown at 5-kb resolution, with dashed lines and open circles marking pairwise contacts between *NMU* promoter, facilitators, and eNMU. Note that some contact anchors may not align perfectly with the regulatory elements, possibly due to the limited resolution of this Hi-C dataset.

(B) Expanded ChIP-seq tracks[3,44] displaying signal p-values. Grey box highlights the *NMU* promoter and its proximal region, with a zoomed-in view shown on the top right. A separate inset on the right zooms in at the eNMU region, shown at the same scale as the full locus, except where otherwise indicated. Detailed sources and accession information are provided in Table S4.

Related to Figure 6.

bioRxiv preprint doi: https://doi.org/10.1101/2025.07.07.663560; this version posted July 9, 2025. The copyright holder for this preprint (which was not certified by peer review) is the author/funder, who has granted bioRxiv a license to display the preprint in perpetuity. It is made available under aCC-BY 4.0 International license.

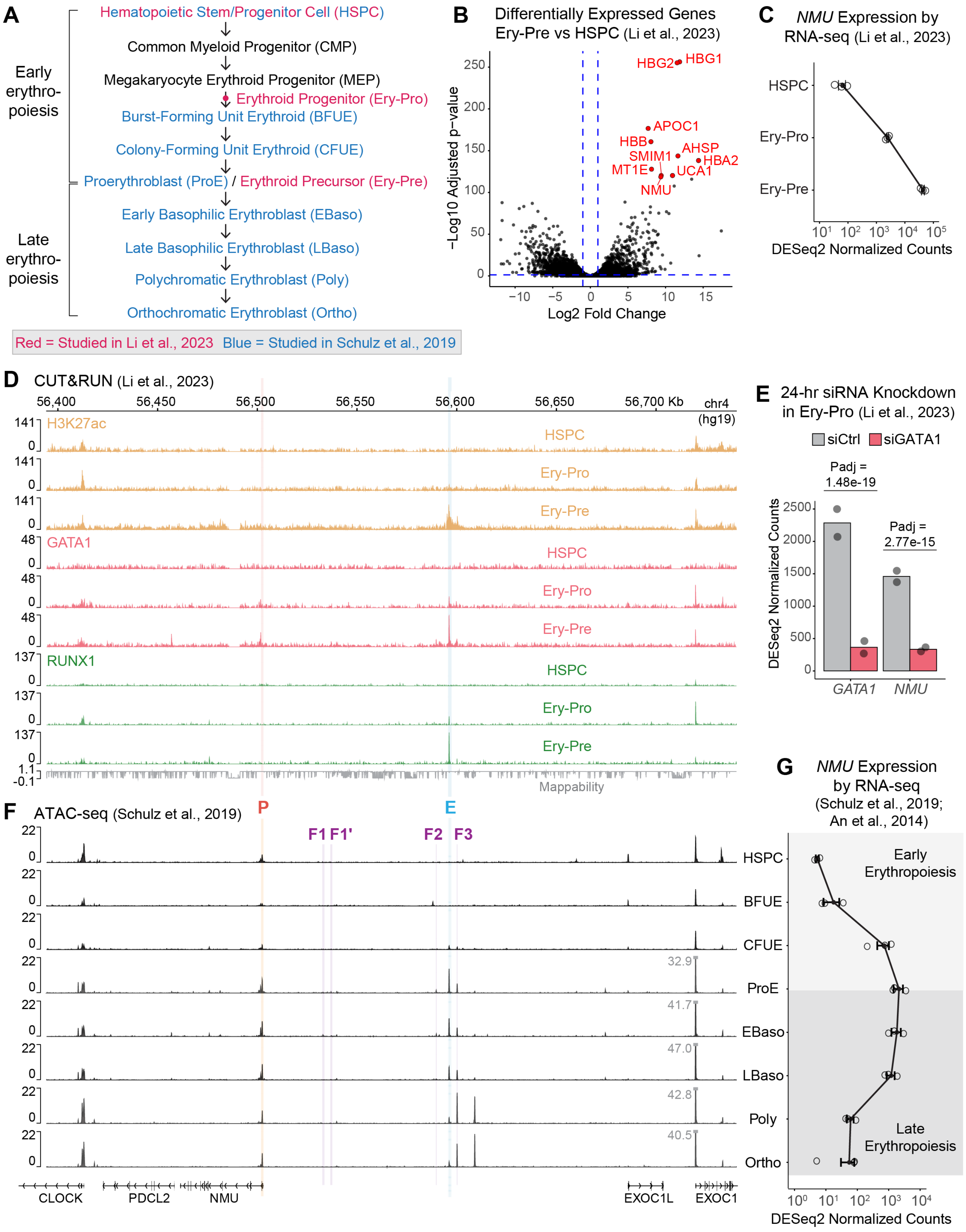

bioRxiv preprint doi: https://doi.org/10.1101/2025.07.07.663560; this version posted July 9, 2025. The copyright holder for this preprint (which was not certified by peer review) is the author/funder, who has granted bioRxiv a license to display the preprint in perpetuity. It is made available under aCC-BY 4.0 International license.

## Figure S7. Dynamics of eNMU regulation during erythroid differentiation

(A) Stages of HSPC erythroid differentiation analyzed in Li et al.[64] (red) and Schulz et al.[66] (blue).

(B) Volcano plot showing genome-wide expression changes between HSPC and Ery-Pre stages, reanalyzed from Li et al.[64] RNA-seq data. Horizontal and vertical blue lines mark adjusted p = 0.05 and $\log_2$ fold changes of ±1, respectively. Red dots highlight the top 10 most significantly upregulated genes, including the key erythroid markers *HBG1* and *HBG2* (β-like globin genes).

(C) *NMU* expression changes during early erythropoiesis, reanalyzed from Li et al.[64] RNA-seq data (n = 3).

(D) CUT&RUN signal of H3K27ac, GATA1 and RUNX1 at the *NMU*–eNMU locus during early erythropoiesis. Tracks show one representative biological replicate from Li et al.[64]

(E) *GATA1* and *NMU* expression changes following 24-hr siRNA knockdown of *GATA1* in Ery-Pro cells, reanalyzed from Li et al.[64] RNA-seq data (n = 2).

(F) ATAC-seq signal at the same locus as in (D) throughout the full HSPC differentiation time course. Tracks show merged biological replicates (n = 2) from Schulz et al.[66]

(G) *NMU* expression changes during the same stages as in (F), reanalyzed from An et al.[65] and Schulz et al.[66] RNA-seq data (n = 3).

Related to Figure 6.
